# The infectivity of the entomopathogenic fungus *Beauveria bassiana *to insecticide-resistant and susceptible *Anopheles arabiensis *mosquitoes at two different temperatures

**DOI:** 10.1186/1475-2875-9-71

**Published:** 2010-03-08

**Authors:** Christophe K Kikankie, Basil D Brooke, Bart GJ Knols, Lizette L Koekemoer, Marit Farenhorst, Richard H Hunt, Matthew B Thomas, Maureen Coetzee

**Affiliations:** 1 School of Animal, Plant and Environmental Sciences, University of the Witwatersrand, Private Bag 3, Wits 2050, Johannesburg, South Africa; 2 Vector Control Reference Unit, National Institute for Communicable Diseases of the National Health Laboratory Service, Private Bag X4, Sandringham 2131, Johannesburg, South Africa; 3 Malaria Entomology Research Unit, School of Pathology, University of the Witwatersrand and the National Health Laboratory Service, Johannesburg, South Africa; 4 Laboratory of Entomology, Wageningen University and Research Centre, PO Box 8031, 6700 EH, Wageningen, the Netherlands; 5 Centre for Infectious Disease Dynamics and Department of Entomology, Pennsylvania State University, University Park 16802, PA, USA

## Abstract

**Background:**

Control of the major African malaria vector species continues to rely extensively on the application of residual insecticides through indoor house spraying or bed net impregnation. Insecticide resistance is undermining the sustainability of these control strategies. Alternatives to the currently available conventional chemical insecticides are, therefore, urgently needed. Use of fungal pathogens as biopesticides is one such possibility. However, one of the challenges to the approach is the potential influence of varied environmental conditions and target species that could affect the efficacy of a biological 'active ingredient'. An initial investigation into this was carried out to assess the susceptibility of insecticide-susceptible and resistant laboratory strains and wild-collected *Anopheles arabiensis *mosquitoes to infection with the fungus *Beauveria bassiana *under two different laboratory temperature regimes.

**Methods:**

Insecticide susceptibility to all four classes of insecticides recommended by WHO for vector control was tested on laboratory and wild-caught *An. arabiensis*, using standard WHO bioassay protocols. Mosquito susceptibility to fungus infection was tested using dry spores of *B. bassiana *under two temperature regimes (21 ± 1°C or 25 ± 2°C) representative of indoor conditions observed in western Kenya. Cox regression analysis was used to assess the effect of fungal infection on mosquito survival and the effect of insecticide resistance status and temperature on mortality rates following fungus infection.

**Results:**

Survival data showed no relationship between insecticide susceptibility and susceptibility to *B. bassiana*. All tested colonies showed complete susceptibility to fungal infection despite some showing high resistance levels to chemical insecticides. There was, however, a difference in fungus-induced mortality rates between temperature treatments with virulence significantly higher at 25°C than 21°C. Even so, because malaria parasite development is also known to slow as temperatures fall, expected reductions in malaria transmission potential due to fungal infection under the cooler conditions would still be high.

**Conclusions:**

These results provide evidence that the entomopathogenic fungus *B. bassiana *has potential for use as an alternative vector control tool against insecticide-resistant mosquitoes under conditions typical of indoor resting environments. Nonetheless, the observed variation in effective virulence reveals the need for further study to optimize selection of isolates, dose and use strategy in different eco-epidemiological settings.

## Background

Malaria vector control relies primarily on the selective application of residual insecticides through either indoor residual house spraying (IRS) or insecticide-treated nets (ITNs). At high coverage, these approaches have proven highly effective in reducing malaria morbidity and mortality at an affordable cost [1]. However, the ever-increasing development of resistance to insecticides [2,3] is of great concern. Insecticide resistance in malaria vector populations covers all classes of insecticides currently used in public health and is widespread geographically [2,4-7]. It is, therefore, not surprising that interest in alternative non-chemical strategies has increased over the last decade.

Fungal pathogens commonly infect insects [8] and there has been extensive research on numerous species of Deuteromycete fungi (e.g. *Culicinomyces spp*., *Beauveria spp*., *Metarhizium spp*. and *Tolypocladium spp*.) for use as biological pest control agents in agriculture [9-13]. Although such fungi appear to have limited impact on mosquito populations under natural conditions [8,14], there is increasing evidence supporting the potential use of isolates of *Beauveria bassiana *and *Metarhizium anisopliae *for control of adult mosquito vectors [15-25].

Given the emerging problems of insecticide resistance, one of the key requirements for any new (bio) pesticide product for mosquito control is to have limited cross-resistance with existing chemical insecticides [26,27]. Clearly, if resistance to widely used insecticides, such as permethrin and DDT, confers resistance to fungal pathogens then potential novel biopesticide products will have limited utility either as replacements for insecticides, or in integrated strategies for insecticide resistance management [18]. The likelihood of cross-resistance occurring in mosquitoes, however, appears to be remote and in fact it would seem that in certain instances infection with fungi counteracts resistance that is based on metabolic mechanisms, at least in laboratory colonies [25]. One aim of the current study, therefore, was to compare the virulence of a candidate strain of the entomopathogenic fungus *B. bassiana *against insecticide-resistant and susceptible *Anopheles arabiensis *laboratory colonies, as well as wild collected adult mosquitoes to determine fungal susceptibility.

Additionally, because fungal pathogens are living organisms, the rate at which they penetrate and grow within an infected host is determined by temperature. Accordingly, the efficacy of certain fungal biopesticide products in agriculture has been shown to be strongly influenced by environmental temperature, in some cases further mediated by thermal behaviour of the target insect [28-31]. The effects can be such that under certain conditions, speed of kill is rapid and overall control very good, while under other conditions, speed of kill is very slow and control inadequate [30,31]. In a recent study, Blanford *et al *[22] demonstrated that *Anopheles stephensi *mosquitoes infected with *B. bassiana *or *M. anisopliae *did not exhibit any change in thermal behaviour that might affect speed of kill. Nonetheless, temperature remains an important environmental factor likely to affect fungal germination and growth rate inside mosquito hosts. With respect to malaria control, a critical factor is how the speed of kill (virulence) varies relative to the extrinsic incubation period (EIP) of the malaria parasite; if mortality is faster than the rate of parasite development then impact on transmission will be greater than if mortality is slower than the parasite rate of development [21]. Importantly, both the EIP and pathogen growth vary with temperature [31,32]. The second aim of the current study, therefore, was to explore the effect of temperature on virulence of *B. bassiana *against insecticide susceptible and resistant *An. arabiensis*. The daily average temperature measured inside traditional African houses between seasons in western Kenya is 23 ± 1.8°C [33-35]. Therefore, the impact of *B. bassiana *was assessed nder temperature regimes 2°C lower and higher than this average to capture the range of mean temperatures likely experienced in indoor resting sites.

## Methods

### Wild mosquito collection

Mosquitoes were collected inside traditional houses in Karonga (9° 48'51.04" S, 33° 52'.97" E), northern Malawi, using a mouth aspirator. They were placed into small polystyrene cups covered with gauze netting and later identified morphologically using the taxonomic keys of Gillies and Coetzee [36]. Only female mosquitoes identified as members of the *Anopheles gambiae *complex were retained. A cohort of these mosquitoes was maintained on a 10% sucrose solution soaked in cotton pads for a few hours after which they were subjected to insecticide susceptibility tests. Another cohort of female mosquitoes was blood-fed for oviposition. These mosquitoes were also maintained on a sucrose solution before and during transportation to the laboratory for colony rearing and fungal susceptibility tests.

### Mosquito rearing

Colonies of *An. arabiensis *housed at the Vector Control Reference Unit of the National Institute for Communicable Diseases, Johannesburg, South Africa, were maintained under standard insectary conditions of 25 ± 2°C, 80% ± 10% relative humidity (RH) and a 12:12 hour day/night cycle with 45 minutes dusk/dawn transition between photo-periods. Field collected mosquitoes were reared under the same conditions in order to obtain F1 progeny to be used for fungal infection experiments. Female mosquitoes were offered blood meals two or three times whereafter each gravid female mosquito was placed in a glass vial lined with a moistened filter paper to allow for egg laying. Individual egg batches were transferred into plastic bowls (250 ml) half filled with distilled water. F1 larvae were reared in the same bowl until they pupated. All larvae were fed twice daily with a mixture of brewer's yeast and finely ground dog biscuits. Pupae were transferred daily into plastic vials (50 ml) half filled with distilled water and covered with gauze netting. Emerged F1 adult mosquitoes were collected using an aspirator and kept in small cups (180 ml) covered with gauze netting or in small plastic cages (2.5 litres). F1 adult progeny were maintained on a 10% sucrose solution soaked in cotton pads for 2-3 days before being subjected to fungal susceptibility tests.

### Species identification

The Polymerase Chain Reaction (PCR) method [37] was used to test the species integrity of selected laboratory-reared *An. arabiensis *colonies as well as to identify wild-caught material morphologically identified as *An. gambiae *complex. Selected laboratory colonies included SENN and SENN-DDT originating from Sudan, and MBN and MBN-DDT originating from Kwazulu/Natal, South Africa. SENN-DDT and MBN-DDT were selected for resistance to DDT from their respective parent colonies.

### Insecticide susceptibility tests

Newly emerged adult mosquitoes from each *An. arabiensis *colony were exposed to discriminating dosages of representative insecticides from all insecticide classes recommended by WHO for malaria vector control (Table 1). The standard insecticide susceptibility assays and test kits were used [38]. Samples of 25 non blood-fed mosquitoes per cylinder were exposed to insecticide-treated papers for one hour. After exposure, mosquitoes were transferred to clean holding tubes and provided with cotton pads soaked in a 10% sucrose solution. Knock-down was recorded after 1 h exposure and final mortality was recorded 24 h post-exposure. Each test was duplicated for fully insecticide susceptible colonies and triplicated for insecticide resistant colonies. Tests to determine the insecticide susceptibility status of field-collected mosquitoes were limited to DDT (organochlorine) and dieldrin (cyclodiene) because of sample size limitations. Insecticide susceptibility status was determined according to WHO criteria, whereby colonies were considered resistant when more than 20% of individuals survived the diagnostic dose 24 h post-exposure. A final mortality of 98-100% indicates full susceptibility whilst mortality between 80-97% suggests the possibility of resistance that needs further confirmation [38].

**Table 1:** Insecticides used for WHO insecticide susceptibility tests.

Class	Insecticide	Diagnostic concentration (%)
Pyrethroids	Deltamethrin	0.05
	Permethrin	0.75
Carbamates	Bendiocarb	0.1
	Propoxur	0.1
Organo-phosphates	Malathion	5
	Pirimiphos-methyl	0.9
Organochlorines	DDT	4
	Dieldrin (cyclodiene)	4

### Fungal susceptibility tests

Fungal infection experiments were performed under two different temperature regimes: 21 ± 1°C and 25 ± 2°C, both under 70% ± 15% relative humidity. Infection was carried out using 'fungal suspensors' prepared according to the method described by Scholte *et al *[15]; this approach is not meant to mimic potential operational delivery of spores but provides a reliable infection method for controlled laboratory-based assays. For each trial three suspensors measuring 6.5 cm in height and 2.5 cm in diameter were lined on the inside with a cylinder of filter paper which also served to hold a plastic vial half filled with a 10% sucrose solution for moistening the filter paper. The sugar solution served as a mosquito attractant to each suspensor. Approximately 100 mg of *B. bassiana *(isolate IMI 391510) conidial powder was weighed and dusted onto treatment suspensors using a small paintbrush. Treated suspensors and an equal number of untreated, control suspensors were placed individually into small cages (2.5 litres). Cohorts of 25-35 sugar-fed 2-3 day old adult female mosquitoes from each colony were exposed to either treated or untreated suspensors for 24 h. A minimum of three trials was conducted per colony, each consisting of three treatment replicates and three controls. After the 24 h exposure mosquitoes were transferred into clean holding cages and fed on a 10% sucrose solution during the monitoring period.

Mortality in treatment and control samples was recorded daily up until the death of all fungus-infected mosquitoes. All dead mosquitoes were removed from their respective cages using dissecting forceps. Each cadaver was dipped in 70% ethanol in order to eliminate saprophytic fungi from their cuticles. They were then placed in Petri dishes lined with moistened filter paper and sealed with parafilm to maintain high humidity for fungal sporulation. Petri dishes were incubated at 25 ± 2°C for a period of 3-5 days after which cadavers were screened for external fungal growth under a compound microscope. Cadavers with visible fungal growth on their body surface were considered to have died as a result of fungal infection. Final fungal infection proportion was recorded per sample.

### Data recording and analysis

Mosquito survival was recorded daily. Cox' regression analysis [39] using SPSS 15.0 software was used to compare survival between treatments and controls. Comparisons of mortality rates were used to assess differences in the effect of fungus infection on survival between insecticide susceptible and resistant colonies, laboratory-reared and wild caught *An. arabiensis *as well as between the two temperature regimes. For each factor, i.e. insecticide susceptibility status, colony and temperature, two-way interactions (of the factor with fungus treatment) were included in the Cox Regression model to test if the effect of fungal infection on mosquito survival was significantly influenced by the factor.

## Results

### Species identification and confirmation

A sample of 386 *An. gambiae *complex mosquitoes was collected from the field in Malawi. They were all identified as *An. arabiensis *by PCR. All samples drawn from the laboratory-reared colonies were confirmed to be as *An. arabiensis*.

### Insecticide susceptibility tests

**Figure 1:**
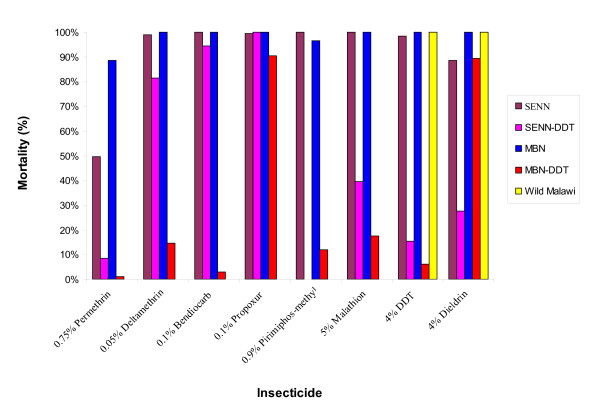
**Insecticide susceptibility status of four laboratory strains and one field strain of *Anopheles arabiensis***. Data show the average mosquito mortality (%) after a 1 hour exposure to insecticide treated papers, recorded 24 hours post-exposure, of two replicates of 25 mosquitoes for the susceptible groups and three replicates for the resistant groups.

The insecticide susceptibility status of the laboratory *An. arabiensis *colonies and the wild-caught sample is shown in Figure 1. There was no evidence of insecticide resistance in MBN and field-collected mosquitoes, although the latter were only exposed to 4% DDT and 4% dieldrin because of limited numbers available for exposure assays. There was clear evidence of resistance to 0.75% permethrin in the baseline colony SENN. The SENN-DDT and MBN-DDT selected colonies showed measurable resistance to DDT as expected, as well as resistance to one or more insecticides from each of the other classes (pyrethroids, carbamates and organophosphates).

### Fungal susceptibility tests

**Figure 2:**
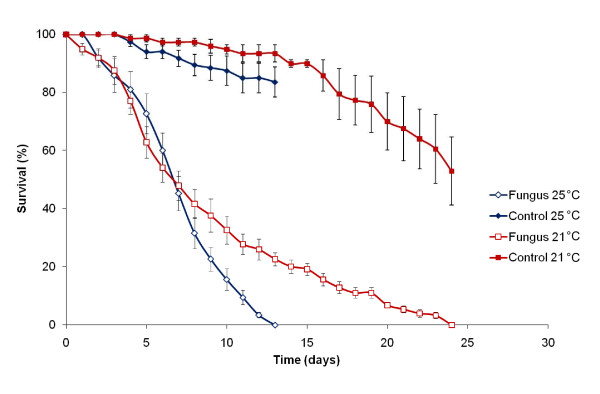
**Daily cumulative proportional survival of F1 offspring of *An. arabiensis *collected in Karonga, Malawi**. Data show the average ± SE survival of nine replicate groups of 25-35 mosquitoes infected with *B. bassiana *(open symbols) and uninfected control groups (closed symbols) kept at 21 ± 1°C (red) or 25 ± 2°C (blue).

**Figure 3:**
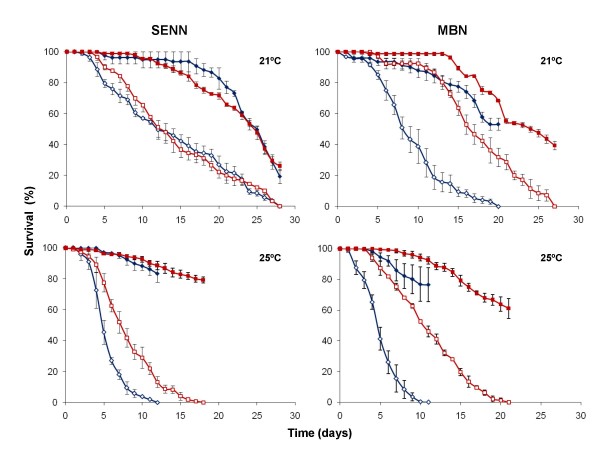
**Daily cumulative proportional mosquito survival of insecticide-susceptible (blue) and insecticide-resistant (red) *An. arabiensis *laboratory colonies originating from Sudan (SENN) or Kwazulu/Natal (MBN)**. Data show the average ± SE survival of nine replicate groups of 25-35 mosquitoes infected with *B. bassiana *(open symbols) and uninfected control groups (closed symbols) kept at 21 ± 1°C (top) or 25 ± 2°C (bottom).

Daily survival of the field-collected mosquitoes after fungus exposure is shown in Figure 2. Exposure to dry *B. bassiana *spores resulted in significant reductions in longevity of the wild *An. arabiensis *mosquitoes (P < 0.05, HR > 1) relative to their respective controls. Comparisons of survival following fungal exposure between wild-caught and baseline laboratory-reared *An. arabiensis *(MBN and SENN, Figure 3) did not reveal any significant difference in susceptibility to fungal infection (P < 0.05, HR > 1).

Survival curves of the insecticide resistant and susceptible laboratory colonies are shown in Figure 3. Exposure to *B. bassiana *spores resulted in significant reductions in longevity in all mosquito colonies (P < 0.05, HR > 1) relative to their respective controls, regardless of their insecticide susceptibility levels and temperature regimes. Cox-regression analysis revealed no significant differences in fungus-infected mortality rates between the insecticide-resistant and baseline colonies. Interaction analyses indicated no significant influence of insecticide susceptibility status on fungus-induced mortality rates at both tested temperatures (HR = 0.769; P = 0.13 and HR = 1.456; P = 0.61 for MBN vs MBN-DDT at 21°C and 25°C respectively and HR = 1.03; P = 0.86 and HR = 0.576; P = 0.27 for SENN vs SENN-DDT respectively at 21°C and 25°C). Even where fungus mortality rates appeared slower in insecticide resistant lines, as in MBN-DDT (Figure 3), this is not because of increased fungal resistance but because the respective untreated control lines also survived better. Therefore, fungal susceptibility is not affected by resistance to insecticides. Fungus-induced mortality rates were relatively rapid at 25°C, with 100% mortality taking 10-12 days post-fungus exposure in the baseline colonies (MBN and SENN) and field-collected mosquitoes, and 18-21 days in the DDT-selected colonies (MBN-DDT and SENN-DDT). At 21°C, equivalent mortalities took 20-28 days and 27-30 days, respectively (Figure 3). The differences in mortality rate were mirrored in the sporulation data, with > 90% sporulation of cadavers in the 25°C regime compared with just over 70% at 21°C. However, not all infected mosquito cadavers show fungal sporulation after death because some mosquitoes may be killed by fungal toxins or secondary infections, rather than primary fungal growth and invasion of organs that tends to facilitate sporulation of the cadaver. As observed from the hazard ratio values in Table 2, the fungus had a stronger effect at higher temperatures than at low temperatures. Cox-regression interaction analyses revealed a significant difference (P = 0.045) in the effect of the fungus on survival between the two temperature regimes. The hazard ratios (risk of death) were greater at the higher temperatures of 25 ± 2°C than at the lower temperatures of 21 ± 1°C (Table 2), suggesting that the probability of a mosquito dying soon after infection was greater at higher temperatures than at lower temperatures. Thus, quantitative differences were detected between the two exposure temperatures in all colonies tested (Figures 2 and 3).

**Table 2:** Mortality P values and hazard ratios of *An. arabiensis *colonies.

	21 ± 1°C		25 ± 2°C	
	
Colony	P value	Hazard ratio	P value	Hazard ratio
MBN	0.001	6.46	0.001	10.23
MBN-DDT	0.001	2.82	0.001	6.99
SENN	0.008	3.13	0.001	23.64
SENN-DDT	0.002	3.20	0.001	16.22
Wild Malawi	0.001	7.62	0.001	16.59

Comparisons were made between fungus-infected and control mosquitoes at 21 ± 1°C and 25 ± 2°C using Cox-regression analysis.

## Discussion

Mosquitoes caught resting indoors north of Karonga on Lake Malawi consisted solely of *An. arabiensis*. Conditions during the collection period were dry and hot, favouring a preponderance of this member of the *An. gambiae *complex, which is generally more tolerant of such conditions [36,40-42].

There are clear indications of insecticide resistance to all insecticides and their respective classes tested in one or more of the *An. arabiensis *samples used. The controlling mechanisms of these resistance phenotypes are likely to involve target site mutations such as *kdr *as well as metabolic detoxification [2,7,43-47]. Resistance to insecticides in major malaria vector species, coupled to the limited number of insecticides available for use in public health programmes, highlights the need to evaluate the potential efficacy of entomopathogens.

All laboratory-reared and wild-caught *An. arabiensis *lines were susceptible to *B. bassiana*. Though quantitative differences were detected between the two exposure temperatures in all colonies tested, *Beauveria *significantly reduced mosquito longevity at both temperature regimes with no evidence for enhanced resistance to fungal infection due to insecticide resistance. Where mortality rate was apparently slowed due to DDT resistance, this effect was due to enhanced overall survival in the selected lines relative to the baseline colonies, rather than any significant reduction in susceptibility to fungus *per se*. Why these resistant mosquitoes survived better than controls in the absence of insecticide exposure is unclear. The DDT resistance in these lines is linked to higher levels of expression of glutathione S-transferases (GST) and esterases [47]. Conceivably these generic detoxifying enzymes could enhance survival in the laboratory environment, although trade-offs against other traits and fitness measures might be expected in other environments [48-50].

*Beauveria *killed mosquitoes significantly quicker at 25°C than at 21°C. This result is consistent with the known temperature-growth profile for this isolate, which indicates a temperature optimum of around 26°C (unpublished data). As suggested previously, to interpret the possible significance of this effect it is important to consider not just absolute speed of kill, but speed of kill relative to the length of the EIP. According to the classic day-degree model of Detinova [51], the EIP of *P. falciparum *is 12.3 days at 25°C and 22.2 days at 21°C. Thus, if mosquitoes became infected with malaria and fungus more or less simultaneously (as would happen if mosquitoes contacted fungus on a treated surface following an infectious blood feed), no mosquitoes would have survived long enough to transmit malaria in any of the colonies held at 25°C. At 21°C, fungal infection of the recently derived Malawi colony would have reduced the percentage of mosquitoes potentially able to transmit malaria (i.e. comparing percent alive at day 22 in control and treated populations) from 64% to 4%, representing a 92% reduction. In the two longer-lived DDT resistant colonies, the equivalent figures are 64% to 17% and 55% to 20%, representing reductions in transmission potential of approximately 70%. Thus, although still contributing to substantial reductions in transmission potential, the fungus appears to work less well at 21°C.

Fully extrapolating these results to potential impact in the field is difficult as mortality schedules could potentially differ markedly between lab and field environments for a variety of reasons. Moreover, the current study considers only one dose and it is likely that higher doses could help compensate for the apparent thermal constraint at 21°C. Studies exploring higher fungal doses and different bioassay exposure techniques have shown the potential for much more rapid mosquito mortality than observed in the current study [e.g. [15,52]], and studies with other insect hosts indicate 'dose x temperature' interactions whereby effects of lower doses are magnified at sub-optimal temperatures [53]. Of course, selection of a different fungal isolate that is less temperature sensitive, or combining isolates with different temperature optima could overcome the constraint completely [18]. Furthermore, the growth rate of the malaria parasite slows exponentially as temperatures decrease further towards 18°C [51], whereas the decline in fungal growth rate appears more linear over this range (unpubl. data) so it is likely that at slightly cooler temperatures still, the relative efficacy of the current isolate would recover. In addition, sub-lethal effects of infection such as impact on malaria parasite development [16,18] and reduced feeding propensity [54,55] can reduce mosquito vectorial capacity irrespective of speed of kill [21]. Nonetheless, with potential for considerable variation in both mean conditions and diurnal temperature ranges across different transmission environments [56], understanding the effects of temperature on biopesticide performance is an important area for further research [see also [57]].

Overall, the results of the current study demonstrate that relative susceptibility of *Anopheles arabiensis *to a candidate fungal biopesticide strain is not affected by resistance to insecticides (see also [25]), that wild-caught mosquitoes are equally susceptible to fungal infection and that although there was temperature-dependent variation in fungal virulence, fungal infection led to substantial reductions in malaria transmission potential in conditions typical of local African houses (at least in western Kenya). These empirical data add support to recent modelling studies suggesting that as long as coverage is high (a goal of most conventional vector control operations), slow acting biopesticides can deliver substantial reductions in malaria transmission across a range of conditions [21,23,58,59]. Of particular relevance here is the study of Koella *et al *[58], who demonstrated that the level of control (whether biological or conventional) necessary to reduce or even prevent malaria transmission depends on the background transmission intensity. In areas of low to moderate transmission, <50% reduction could provide substantial control, whereas in areas of very high transmission, even 90% reduction might not be sufficient to deliver any benefit due to the strongly saturating relationship between malaria prevalence and transmission [58,60]. Thus the significance of the temperature-dependence in biopesticide performance needs to be considered in relation to the local epidemiology context.

Additionally, the efficacy and impact of a biopesticide will depend on ultimate use strategy. For example, in a recent theoretical study, Hancock [59] demonstrated that under conditions of intense transmission, high single coverage of either ITNs or IRS with a fungal biopesticide might not substantially reduce malaria prevalence in the human population, whereas intermediate coverage of both interventions simultaneously could. This conclusion, together with the empirical data demonstrating that resistance to insecticides does not confer resistance to fungi, highlights the potential for development of novel integrated control strategies combining insecticide and biopesticide interventions. Understanding such interactions, together with the local environmental context, are important areas for future research to define possible limits to biopesticide performance and identify isolates, doses and potential delivery systems to optimise control strategies across time and space.

## Competing interests

The authors declare that they have no competing interests.

## Authors' contributions

CKK carried out wild mosquito collections, species identification, insecticide and fungal susceptibility tests, data analysis, interpretation of results, and drafted the first version of the manuscript. BDB supervised all the laboratory experiments and contributed to the subsequent writing of the manuscript. BGJK obtained funding for the project and contributed to the editing of the manuscript. LLK supervised the insecticide susceptibility assays and species identification. MF assisted with the statistical analyses and contributed to the editing of the manuscript. RHH organised the field trip to Malawi and was involved in wild mosquito collections, identification and rearing. MBT provided fungal spores, was involved in methodology of fungal experiments and contributed to editing the manuscript. MC was involved in project design and contributed to the final editing of the manuscript. All authors read and approved the final version of the manuscript prior to submission.
